# Impaired insula functional connectivity associated with persistent pain perception in patients with complex regional pain syndrome

**DOI:** 10.1371/journal.pone.0180479

**Published:** 2017-07-10

**Authors:** Jae-Hun Kim, Soo-Hee Choi, Joon Hwan Jang, Do-Hyeong Lee, Kyung-Jun Lee, Won Joon Lee, Jee Youn Moon, Yong Chul Kim, Do-Hyung Kang

**Affiliations:** 1 Department of Radiology, Samsung Medical Center, Sungkyunkwan University School of Medicine, Seoul, Republic of Korea; 2 Department of Psychiatry, Seoul National University Hospital, Seoul, Republic of Korea; 3 Department of Psychiatry and Institute of Human Behavioral Sciences, Seoul National University College of Medicine, Seoul, Republic of Korea; 4 Department of Medicine, Seoul National University College of Medicine, Seoul, Republic of Korea; 5 Department of Anesthesiology and Pain Medicine, Seoul National University Hospital, Seoul, Republic of Korea; Southeast University Zhongda Hospital, CHINA

## Abstract

Given that the insula plays a contributory role in the perception of chronic pain, we examined the resting-state functional connectivity between the insular cortex and other brain regions to investigate neural underpinnings of persisting perception of background pain in patients with complex regional pain syndrome (CRPS). A total of 25 patients with CRPS and 25 matched healthy controls underwent functional magnetic resonance imaging at rest. With the anterior and posterior insular cortices as seed regions, we compared the strength of the resting-state functional connectivity between the two groups. Functional connectivity between the anterior and posterior insular cortices and the postcentral and inferior frontal gyri, cingulate cortices was reduced in patients with CRPS compared with controls. Additionally, greater reductions in functional connectivity between the anterior insula and right postcentral gyrus were associated with more severe sensory pain in patients with CRPS (short-form McGill Pain Questionnaire sensory subscores, *r* = -.517, *P* = .023). The present results imply a possible role of the insula in aberrant processing of pain information in patients with CRPS. The findings suggest that a functional derangement of the connection between one of the somatosensory cortical functions of perception and one of the insular functions of awareness can play a significant role in the persistent experience of regional pain that is not confined to a specific nerve territory.

## Introduction

Complex regional pain syndrome (CRPS) is characterized by persisting pain and related symptoms such as allodynia and hyperalgesia, trophic changes, neurovascular abnormalities, and motor dysfunction [1,2]. An estimated 50,000 new cases of CRPS with devastating impact on daily functioning are reported in the United States each year [3]. CRPS is accompanied by ongoing chronic pain with maladaptive changes in the functional organization of the primary sensorimotor cortex (S1/M1) including contraction of the cortical representation of the affected limb and simultaneous expansion of cortical representation of the non-affected limb [4–6]. Moreover, the clinical impacts of CRPS include emotional disturbance [7], neurocognitive dysfunction including parietal lobe-related praxis [8], and working memory impairment [9,10].

The underlying pathophysiology of CRPS, which is characterized by continuous chronic background pain and intermittent aggravation of pain intensity [1] can be investigated using resting-state functional connectivity networks (rs-FCN) of spontaneous brain activity as an indicator of functional crosstalk among brain regions. The rs-FCN analysis maps temporally synchronous, spatially distributed fluctuations in regional blood-oxygen level-dependent (BOLD) signals [11–13]. Previous studies using the rs-FCN approach in patients with CRPS have demonstrated increased amygdala-centered functional connectivity with cortical and subcortical regions [7] and reduced functional connectivity in the default mode network [14]. Moreover, previous studies found that the functional connectivity map of the S1/M1 was more diffuse in patients with CRPS than in healthy controls and that reduced connectivity between the S1/M1 and posterior insula was associated with higher spontaneous pain levels [14]. In contrast, Baliki *et al*. [15] found that pain intensity was positively associated with the functional connectivity between the medial prefrontal cortex and insula.

Recent anatomical and functional neuroimaging studies have provided evidence of structural changes in the brain associated with CRPS-related clinical symptomatology as well as alterations in the strength of functional connectivity between cortical regions including the somatosensory cortex, insula, anterior cingulate cortex (ACC), and dorsolateral/dorsomedial prefrontal cortex and subcortical structures such as the hippocampus, amygdala, basal ganglia, and thalamus [7,16–21]. Of note, the gray matter volume [22] and density [23] of the insula, a limbic region that relays and integrates bodily sensations, such as pain, and feelings of emotion [24–26] are decreased in patients with CRPS. Previous studies have shown that the insula has reciprocal anatomical connections with multiple sensory, motor, limbic, and association areas [27,28]. Thus, insula-related changes in brain function may have a significant effect on the interoceptive representation of pain in CRPS.

Few studies have investigated the role of the insula in the pathophysiology CRPS from the perspective of functional connectivity. Additionally, although the subdivisions of the insula have shown different functional organizations [29], there were no attempts to investigate the role of subregions of the insula in CRPS. We aimed to examine the rs-FCN between the anterior/posterior insular cortices and other brain regions to investigate neural underpinnings of continuous perception of chronic background pain in patients with complex regional pain syndrome. We hypothesized that the aberrant functional connectivity of the insula with the limbic or somatosensory cortices would be associated with affect or sensory pain severity in patients with complex regional pain syndrome.

## Materials and methods

### Participants and measurements

Twenty-five patients with CRPS were recruited from the Pain Clinic at Seoul National University Hospital from February 2011 to February 2012. All patients were diagnosed by a board-certified anesthesiologist, based on the International Association for the Study of Pain diagnostic criteria [30]. Pain severity was assessed using the short-form McGill Pain Questionnaire (MPQ) [31]. The MPQ consists of 15 word descriptors of pain (11 sensory and 4 affective), which are rated on an intensity scale of 0–3. The pain scores are derived from the sum of the sensory (range, 0–33) and affective (range, 0–12) intensity ratings. A visual analog scale was used to measure present pain severity on a 10-cm line anchored at the left with ‘‘no pain” and at the right with ‘‘worst possible pain”. Functional level was assessed using the Global Assessment of Functioning (range, 0–100). The psychiatric comorbidities of patients with CRPS were evaluated by a psychiatrist using the Structured Clinical Interview for DSM-IV Axis I Disorders [32]. The 21-item Beck Depression Inventory (BDI; range, 0–63) [33] and the Beck Anxiety Inventory (BAI, range, 0–63) [34] were administered to evaluate the severity of depression and anxiety, respectively. Patients were asked not to change their medications before undergoing the magnetic resonance imaging (MRI) scan. A total of 25 healthy control subjects with no lifetime history of psychiatric disorders or hospitalization, and who were not taking any medications, were recruited via internet advertisement. The exclusion criteria included a known history of substance abuse or dependence, neurological disease or brain injury, or evidence of a medical illness that could cause psychiatric symptoms. The participants took part in our previous study, using a different methodology, to investigate hypotheses different from those involved in the present study [35]. Our study was approved by the Institutional Review Board of Seoul National University Hospital, and written informed consent was obtained from all participants. This study was conducted in accordance with the Declaration of the World Medical Association.

### Functional imaging data acquisition and analysis

All data were acquired using a 3.0-Tesla MRI scanner (Siemens Magnetom Trio, Erlangen, Germany). For each participant, we collected 116 contiguous echo planar imaging functional volumes (time repetition [TR] = 3,500 ms; time echo [TE] = 30 ms; flip angle = 90°; 35 slices; matrix = 128 × 128; field of view [FOV] = 240 × 240 mm^2^; acquisition voxel size = 1.88 × 1.88 × 4.2 mm^3^). Complete cerebellar coverage was obtained for all participants. Participants were explicitly asked to close their eyes, relax, and refrain from focusing on any particular thought during the scan. For spatial normalization, localization and definition of the insular cortex, a high-resolution sagittal T1-weighted anatomical image was acquired using a 3D MPRAGE sequence (TR = 1670 ms; TE = 1.89 ms; flip angle = 9°; 208 slices; FOV = 240 × 240 mm^2^; acquisition voxel size = 0.98 × 0.98 × 1.0 mm^3^).

Data pre-processing was performed using the fMRIB software library (FSL) (www.fmrib.ox.ac.uk). After discarding the first five images to eliminate any signal decay, preprocessing was performed including slice-timing correction, head motion correction, and temporal scaling to yield a whole-brain mode value of 1000. Functional connectivity analysis was performed using the Analysis of Functional NeuroImages (AFNI, http://afni.nimh.nih.gov/afni/). Data were temporally band-pass filtered (0.009–0.08 Hz) using *3dFourier* and spatially smoothed (5-mm full width at half maximum Gaussian blur) using *3dmerge*. Several sources of the spurious variance along with their temporal derivatives were then removed from the data through linear regression using *3dDeconvolve*: six parameters obtained by rigid body correction of head motion, the signal from a ventricular region of interest, and the signal from a region centered in the white matter. The spatial normalization was performed by using a high resolution T1-weighted MR image.

The functional anterior/posterior insular network was made up of brain areas that were positively correlated with the manually defined anterior/posterior insular seed. We used regions-of-interest (ROIs) of the insula that were prepared in advance by our group. The anterior and posterior insular regions were manually delineated on the high-resolution T1-weighted MR images of 33 healthy normal subjects who were not included in the present study (S1 Fig). The manually defined anterior and posterior ROIs were normalized to MNI space. The normalized ROIs were averaged across the 33 subjects and thresholded to ensure 50% probability for each anterior and posterior insular ROI to extract the robust seed mask for the functional connectivity analysis (S2 and S3 Figs). MNI coordinates were [-38 mm, 6 mm, -2 mm] for the left anterior insular ROI, [-36 mm, -18 mm, 6 mm] for the left posterior insular ROI, [40 mm, 2 mm, 2 mm] for the right anterior insular ROI, and [40 mm, -12 mm, 6 mm] for the right posterior insular ROI. For each subject, the representative time series for the anterior/posterior insular regions were obtained by averaging the residual time series over all the voxels in the anterior/posterior insular ROIs using *3dmaskave*. Functional connectivity maps were created by computing the Pearson’s correlation coefficients of the time courses for the anterior/posterior insular ROIs from the time course for all gray matter voxels using *3dfim+*. The correlation coefficients were normalized using Fisher’s z transform using *3dcalc*.

### Statistical analysis

Based on our previous rs-FCN study [36], we performed a power analysis to determine a sample size with the effect size at 1.412, an alpha error probability at 0.001, and the statistical power at 0.8. The sample of the present study was sufficient to perform because the minimum number of each group was 20. Two-sample *t*- and Chi-square tests were used to compare between-group differences in demographic and clinical characteristics. Pearson’s correlation coefficients were calculated to investigate the relationship between symptom severity in patients with CRPS and the strength of functional connectivity with the insula. Statistical analyses were two-tailed, with a significance level of 0.05.

The within-group imaging analysis was performed using a one-sample *t*-test using a *q* < 0.01 false-discovery-rate (FDR) correction threshold. A permutation test was performed to detect significant differences in the degree of functional connectivity between groups. In the permutation test, 50 subjects were randomly assigned to one of two groups, and the distribution of group differences was estimated based on 5000 randomized iterations for each voxel. The voxels, reaching 0.5% (*P* < 0.005, two-tailed) of the estimated distribution of group differences were deemed statistically significant. Clusters less than 64 mm^3^ (about 4 voxels in original space) were excluded.

## Results

### Demographic and clinical characteristics of participants

The demographic and clinical characteristics of the participants are shown in Table 1 and S1 Table. Age (the patient group, rage = 20–59, median = 36 year; the control group, range = 24–48, median = 31 year; 95% Confidence Interval of the difference, -0.949, 9.749), sex, and handedness were not significantly different between the patients and healthy subjects. Most of patients were diagnosed with CRPS type I (92%), had psychiatric comorbidities (76%), and were taking various medications, including opioids (*N* = 13), nonsteroidal anti-inflammatory drugs (*N* = 12), anticonvulsants (*N* = 23), antidepressants (*N* = 20), antipsychotics (*N* = 10), and anxiolytics (*N* = 19). The patients’ BDI and BAI scores were higher than those of the healthy controls. According to the standardization studies on Korean population [37,38], 16 and 13 patients with CRPS showed higher scores than the cutoff values (respectively on the BDI = 21 and BAI = 22). Therefore, in the following results, we additionally considered the potential influence of psychiatric comorbidities of depression and anxiety by using sub-group comparison and partial correlation analyses with covariates of the BDI and BAI scores in the patient group.

**Table 1:** Demographic and clinical characteristics of the study participants.

Variables	Patients with CRPS (*N* = 25)	Healthy controls (*N* = 25)	*χ*^*2*^ or *t*	*P*
Male, n (%)	12 (48)	14 (56)	0.32	0.571
Right handedness, n (%)	23 (92)	22 (88)	0.22	0.637
CRPS type I/II, n	23/2	-	-	-
Initial pain location, n (%)				
Upper limb	6 (24)	-	-	-
Lower limb	6 (24)	-	-	-
Multiple sites	13 (52)	-	-	-
Psychiatric comorbidity, n (%)				
Major depressive disorder	11 (44)	-	-	-
Other mood disorders	7 (28)	-	-	-
Anxiety disorder	1 (4)	-	-	-
	**Mean (SD)**	**Mean (SD)**		
Age, year	36.1 (11.4)	31.7 (6.7)	1.66	0.104
Duration of illness, year	2.8 (3.0)	-	-	-
McGill Pain Questionnaire				
Sensory^a^	19.9 (9.8)	-	-	-
Affect^b^	6.9 (3.7)	-	-	-
Visual analogue scale^c^	5.0 (2.3)	-	-	-
Global Assessment of Functioning	51.1 (14.4)	-	-	-
Beck Depression Inventory^c^	28.0 (13.7)	3.0 (2.9)	8.57	< 0.001
Beck Anxiety Inventory^c^	29.1 (16.1)	2.3 (3.1)	7.87	< 0.001

Abbreviations: CRPS, complex regional pain syndrome.

^a^ *N* = 19,

^b^ *N* = 19,

^c^ *N* = 23.

### Rs-FCN of the anterior insula

**Fig 1:**
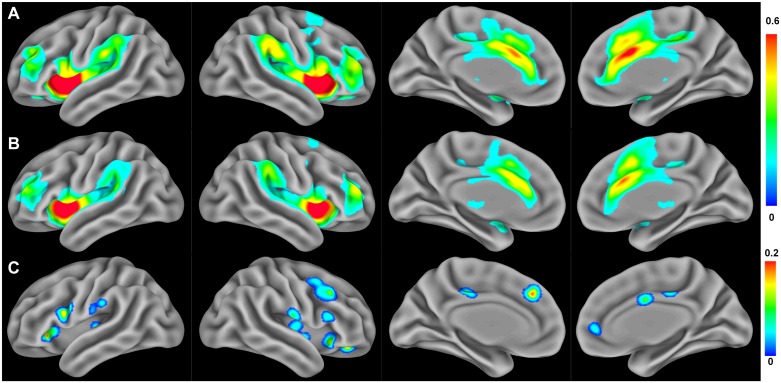
Resting state functional connectivity networks of the anterior insula. (A) Functional connectivity patterns in healthy controls and (B) patients with CRPS (FDR < 0.01, within-group analysis). (C) Brain areas showing a significant reduction in anterior insula functional connectivity in patients with CRPS relative to healthy controls (*P* < 0.005 for permutation test, between-group analysis).

The within-group analysis revealed that the bilateral anterior insular cortices were functionally connected to a wide range of subcortical and cortical areas in healthy controls, including the bilateral thalamus, posterior insula, superior/middle/inferior frontal cortices, pre/postcentral gyri, anterior/posterior cingulate cortices, parietal cortex, and supplementary motor area (Fig 1A), whereas restricted spatial extension and connectivity strengths were observed in the patients with CRPS (Fig 1B).

The between-group analysis revealed that the rs-FCN of the anterior insula was reduced in the bilateral posterior insular cortices, bilateral postcentral and inferior frontal gyri, anterior/posterior cingulate cortices, dorsomedial prefrontal cortex, and right supplementary motor area in patients with CRPS compared with healthy controls (Fig 1C and Table 2). When compared the sub-groups according to the psychiatric comorbidities of depression or anxiety in the patient group, these brain regions had no significant differences in the strength of rs-FCN with the anterior insula. There were no brain regions showing increased rs-FCN with the anterior insula in patients with CRPS than healthy controls.

**Table 2:** Brain regions showing decreased functional connectivity with the anterior and posterior insular cortices in patients with CRPS relative to healthy control subjects.

Brain region, Brodmann area	MNI Coordinates			Volume (mm^3^)	Max intensity
	x	y	z		
***Anterior insula***					
B posterior insula, 13	40	-6	-6	496	0.191
	36	-10	6	184	0.191
	-34	-18	4	592	0.237
B postcentral gyrus, 1	64	-14	18	360	0.259
	-64	-18	22	576	0.225
B inferior frontal gyrus, 45/47	56	20	20	1,520	0.252
	-56	12	26	808	0.251
	32	24	-10	784	0.236
	-36	26	-2	560	0.273
Anterior cingulate cortex, 32/24	6	50	2	520	0.273
	4	2	34	176	0.214
Posterior cingulate cortex, 31	8	-24	40	160	0.195
	-6	-24	38	328	0.204
Dorsomedial prefrontal cortex, 8	-8	36	38	288	0.262
R supplementary motor area, 6	40	10	52	176	0.190
***Posterior insula***					
B anterior insula	40	2	4	880	0.223
	36	12	-4	296	0.165
	-34	6	-8	336	0.217
R postcentral gyrus, 2	56	-28	46	352	0.219
R inferior frontal gyrus, 45/47	44	12	22	296	0.206
	28	22	-10	296	0.186
	46	26	6	312	0.235
Posterior cingulate cortex, 31	12	-38	44	672	0.262
Dorsomedial prefrontal cortex, 8	-4	32	50	216	0.196

Abbreviations: CRPS, complex regional pain syndrome; B, Bilateral; R, Right; L, Left.

### Rs-FCN of the posterior insula

**Fig 2:**
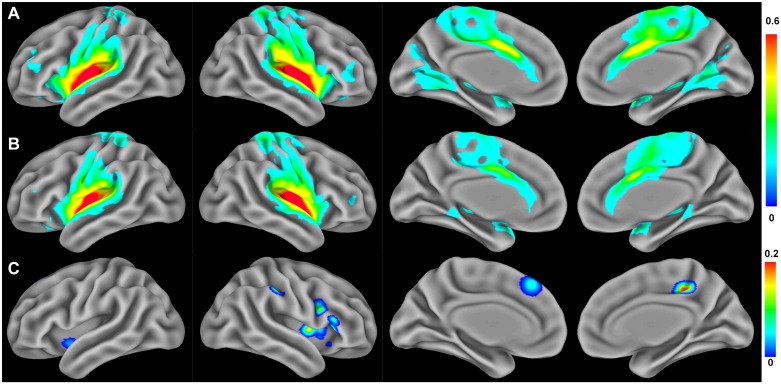
Resting-state functional connectivity networks of the posterior insula. (A) Functional connectivity patterns in healthy controls and (B) patients with CRPS (FDR < 0.01, within-group analysis). (C) Brain areas showing a significant reduction in posterior insula functional connectivity in patients with CRPS relative to healthy controls (*P* < 0.005 for permutation test, between-group analysis).

The within-group analysis revealed that the bilateral posterior insular cortices were functionally connected to the bilateral thalamus, anterior insula, middle/inferior frontal cortices, pre/postcentral gyri, lingual gyrus, precuneus, anterior/posterior cingulate cortices, and supplementary motor area in healthy controls (Fig 2A). However, these connectivity patterns were shown to be limited in patients with CRPS (Fig 2B).

The between-group comparison of the posterior insula rs-FCN revealed that functional connectivity was significantly decreased in patients with CRPS in the bilateral anterior insular cortices, right postcentral and inferior frontal gyri, posterior cingulate, and dorsomedial prefrontal cortices (Fig 2C and Table 2). When compared the sub-groups according to the psychiatric comorbidities of depression or anxiety in the patient group, these brain regions had no significant differences in the strength of rs-FCN with the posterior insula. There were no brain regions showing increased rs-FCN with the posterior insula in patients with CRPS than healthy controls.

### Correlations between symptom severity and rs-FCN strength in the insula

**Fig 3:**
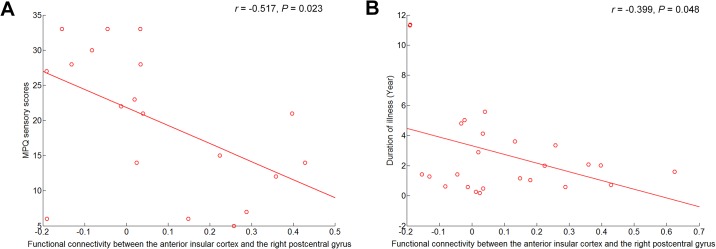
Scatter plots showing the correlations of functional connectivity in the anterior insula and right postcentral gyrus with (A) pain severity and (B) illness duration. Abbreviations: MPQ, McGill Pain Questionnaire.

The correlation analyses revealed that the strength of the anterior insula and right postcentral gyrus connectivity was inversely correlated with MPQ sensory scores (*r* = -0.517, *N* = 19, *P* = 0.023) and duration of illness (*r* = -0.399, *N* = 25, *P* = 0.048) in patients with CRPS (Fig 3). In the partial correlation analyses using covariates of the BDI and BAI scores, these correlations remained meaningful (MPQ sensory scores, *r* = -0.553, *df* = 15, *P* = 0.021; duration of illness, *r* = -0.394, *df* = 19, *P* = 0.078). Other than the correlations listed above, there were no additional associations.

## Discussion

We found that the functional connectivity of the anterior and posterior insular cortices with the postcentral and inferior frontal gyri, cingulate and dorsomedial prefrontal cortices was reduced in patients with CRPS. Furthermore, reduced functional connectivity between the anterior insula and right postcentral gyrus was associated with increased perception of severe pain in patients with CRPS, suggesting that a disconnection between the somatosensory cortical function of perception and insular function of awareness or regulation may play a significant role in persistent regional pain that is not confined to a specific nerve territory or dermatome.

A previous population-based cohort study found that the majority of patients with CRPS reported persistent impairment 2 or more years after pain onset [39]. Most of our patients (23 of 25) had also chronic CRPS (> 6 months duration). A recent study found that the local accumulation of mast cells was increased in acute (< 3 months) but not in chronic CRPS (> 3 months) [40]. Thus, CRPS symptoms lasting longer than 3–6 months can no longer be explained by peripheral pathophysiology [2], suggesting that after the initial inflammation, other central mechanisms contribute to the perception of persistent pain. One such pathophysiological mechanism may involve brain plastic changes in response to increased input from peripheral nociceptors [41]. Central sensory reorganization of the primary somatosensory cortex in CRPS has been widely investigated [6]. However, the reality that pain that does not correspond to a dermatome and may spread beyond the initially affected limb in patients with CRPS suggests an aberration in the interoceptive perception of pain rather than a localized abnormality in the somatosensory cortex. The insula, which is involved in the awareness of pain [24,25], can play a crucial role in the perception of continuous pain in patients with CRPS. Our finding of reduced functional connectivity between the anterior and posterior insular cortices and the pain-related sensory and affective regions in patients with CRPS supports this role of the insula.

Head and Holmes [42] hypothesized that central pain was a release phenomenon resulting from the loss of an inhibitory effect of discriminative pain processing on the emotional aspect of pain [24]. Similarly, persistent pain associated with CRPS may result from the loss of descending control of the interoceptive function of the insula on the sensory system. The insula is among the cortical regions showing structural abnormalities in patients with CRPS [22]. The postcentral gyrus (primary somatosensory cortex) has been implicated in processing of pain as well as sensory information [43], and a reduction in the spatial representation of the CRPS-affected limb in the somatosensory cortex has been reported previously [6]. We found that the reduced functional connectivity between the anterior insula and postcentral gyrus was associated with an increase in the perceived intensity of the sensory component of pain in patients with CRPS. The anterior insula is functionally connected with cognitive and affective processing areas while the posterior insula is connected with the sensorimotor processing areas [29], and our within-group results (Figs 1 and 2) are also in line with these previous findings. Thus, the functional connection of the cortical regions with the posterior insula seems to correlate with actual pain intensity, while that of the anterior insula seems to be related to the perceived pain intensity. The anterior insula would pass this information to the higher systems involved in the top-down attention and cognitive control mechanism of pain. Our correlation finding suggests that insufficient reciprocal communication between the anterior insula and somatosensory cortex may contribute to disproportionate perception and dysfunctional regulation of pain in CRPS.

A previous study found that when the S1/M1 was defined as the ROI, the functional connectivity between the S1/M1 and the right insula, posterior cingulate cortex, superior parietal lobule, and motor areas was stronger in the patients with CRPS than in healthy controls [14]. Although our findings do not agree with this aspect of the Bolwerk *et al*. study, their finding of a negative correlation between pain intensity and functional connectivity between S1/M1 and the posterior insula in patients with CRPS, suggesting impaired pain modulation, is consistent with that of ours. Moreover, the present results revealed that the longer the duration of the illness, the greater the reduction in functional connectivity between the anterior insular cortex and the postcentral gyrus. Our findings are consistent with a previous study that found an association between anterior insular atrophy and CRPS intensity and duration [44]. This association suggests that the reduced functional connectivity between the anterior insula and the postcentral gyrus may be an aftereffect of illness duration.

Furthermore, Craig et al. demonstrated a posterior-to-mid-to-anterior progression in the integration of interoceptive information in the insula [25,45]. Our finding of a decrease in the functional connectivity between the anterior and posterior insular cortices in patients with CRPS suggests that the progression of the integration of interoceptive representations is disrupted, which may impair the ability to differentiate between noxious and innocuous stimuli (e.g., allodynia). Indeed, a previous study found that the insula mediated allodynia in patients with CRPS along with the somatosensory cortex and ACC [46]. In a pain provoking study in patients with anorexia nervosa, the left posterior insula reflected increased pain thresholds in patients, while increased activations in the right anterior insula and pons might mirror increased adrenergic descending pain inhibition [47]. Thus, the anterior and posterior insular cortices seem to play complementary roles for the same pain stimuli.

The inferior frontal gyrus and ACC are anatomically and functionally connected with the insula [25,48]. Especially, the ventrolateral part of the inferior frontal gyrus (Brodmann area 47) of our findings is correspondent to the fronto-insular cortex. The fronto-insular cortex and ACC have been shown to play prominent roles in the switching between activation of higher-order cognitive networks and deactivation of task-irrelevant networks during cognitively demanding tasks [48]. Furthermore, the fronto-insular cortex and ACC play critical roles in the interoceptive awareness of stimulus-induced and stimulus-independent changes [24,48,49]. The ACC is involved in processing the affective aspect of pain perception [50] and is implicated in chronic pain [51]. Thus, reduced functional connectivity between these regions and the insula may impair the processing of interoceptive awareness and contribute to the maladaptive persistent pain characteristic of CRPS. The right inferior frontal cortex is involved in inhibitory control and is turned on endogenously for self-control rather than externally generated control [52,53]. Therefore, reduced communication between the inferior frontal cortex and insula may contribute to the dysfunctional endogenous inhibition or regulation of pain in patients with CRPS.

Although their prevalence does not seem to be higher than in other chronic pain patients [54], it deserves to mention that psychiatric comorbidities, such as depression and anxiety, are common in patients with CRPS [55]. In our clinical sample, considerable numbers of CRPS patients (66.7%) have been reported to have depressive or anxiety disorders [56]. In the current study, 69.6% or 56.5% of patient with CRPS were also shown to have depression or anxiety, respectively. These comorbidities might be associated with the pathophysiology of central mechanism or sympathetic nervous system in CRPS [57]. However, our additional statistical analyses for controlling the potential confounding effects of psychiatric comorbidities revealed that the current results were not affected by mood-related changes. Furthermore, the previous study that excluded patients with psychiatric comorbidities also reported the insular pathology in CRPS [44].

Our study has several limitations. First, all of our patients were taking medications that acted on the central nervous system, which may have had an impact on the functional structure of the brain during rest. Future studies are needed to investigate whether the use of opioids or other analgesics and psychotropic drugs influence the rs-FCN in patients with CRPS. However, scanning the patients who are not on medication may display a transient state of the perception of intense pain. Second, the heterogeneity of our patient population (e.g., various initial pain locations and psychiatric comorbidities) may have influenced our results. Further studies with larger populations and subgroup analyses are needed to clarify the impact of patient heterogeneity. Third, our study design did not allow us to investigate causality, i.e., the role of impaired resting-state functional connectivity of the insula on the development of CRPS. An investigation of the progression of the illness and CRPS subtypes [57] is needed to address this issue. Fourth, there were no behavioral measures of aberrant pain perception and persistent awareness of pain. The present study simply assumed that related clinical features of CRPS, such as allodynia, hyperalgesia, body perception disturbances, and continuing pain, would be expressed as a pain severity in patients. Thus, the present approach precludes an inference of a direct relationship between behavioral and neural correlates. Fifth, since we grouped patients with the right and left lesions, the present study could not explore the functional lateralization in the pathology of CRPS. Further research is needed to investigate the lesion-specific pathology on the left-right lateralization or asymmetry in rs-FCN of the somatosensory cortices with a large population. Finally, relatively liberal, uncorrected significance levels were used for the correlation analyses.

In summary, impaired functional connectivity of the insula with the postcentral and inferior frontal gyri and cingulate cortices in patients with CRPS can be associated with a persistent awareness of pain. In particular, alterations in the rs-FCN between the anterior insula and postcentral gyrus were associated with the sensory perception of pain in patients with CRPS. As a key region of interoceptive perception of pain, the insula may play a central role in aberrant processing of pain information in patients with CRPS.

## Supporting information

S1 Fig Manually defined anterior and posterior insular cortices on the T1-wieghted MRI in the left hemisphere (A) and in the right hemisphere (B). Orange, anterior insular cortex in the left hemisphere; Red, posterior insular cortex in the left hemisphere; Green, anterior insular cortex in the right hemisphere; Yellow, posterior insular cortex in the right hemisphere.
(TIF)

S2 Fig ROIs for anterior and posterior insular cortices in the left hemisphere. (A) Probability map for the anterior insular cortex from 33 healthy subjects. (B) Anterior insular to ensure 50% probability in (A). (C) Probability map for the posterior insular cortex from 33 healthy subjects. (D) Posterior insular to ensure 50% probability in (C).
(TIF)

S3 Fig ROIs for anterior and posterior insular cortices in the right hemisphere. (A) Probability map for the anterior insular cortex from 33 healthy subjects. (B) Anterior insular to ensure 50% probability in (A). (C) Probability map for the posterior insular cortex from 33 healthy subjects. (D) Posterior insular to ensure 50% probability in (C).
(TIF)

S1 Table Participant characteristics. (DOCX)
