# Supplementary figures and images for: Impaired insula functional connectivity associated with persistent pain perception in patients with complex regional pain syndrome
Source: PLoS One. 2017 Jul 10;12(7):e0180479. doi: 10.1371/journal.pone.0180479 (PMC5503260; doi:10.1371/journal.pone.0180479)

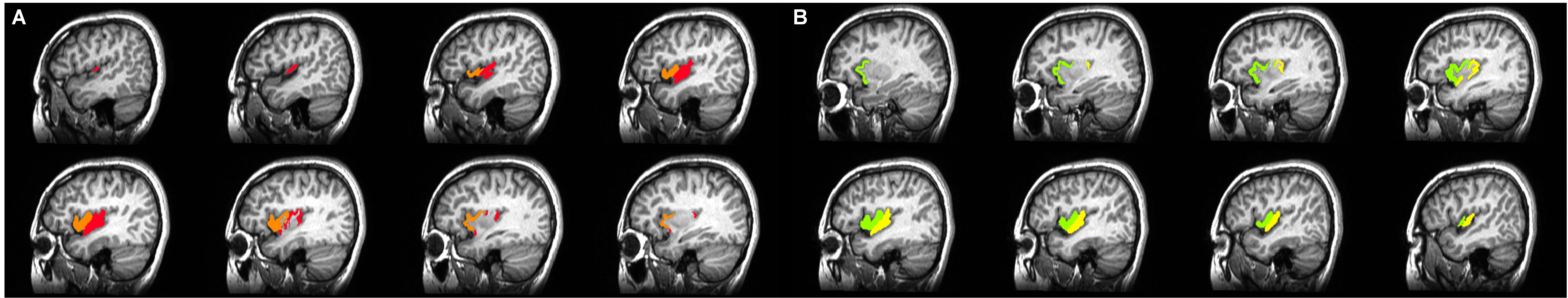

Supplement: S1 Fig — Orange, anterior insular cortex in the left hemisphere; Red, posterior insular cortex in the left hemisphere; Green, anterior insular cortex in the right hemisphere; Yellow, posterior insular cortex in the right hemisphere. (TIF) [file pone.0180479.s001.tif]

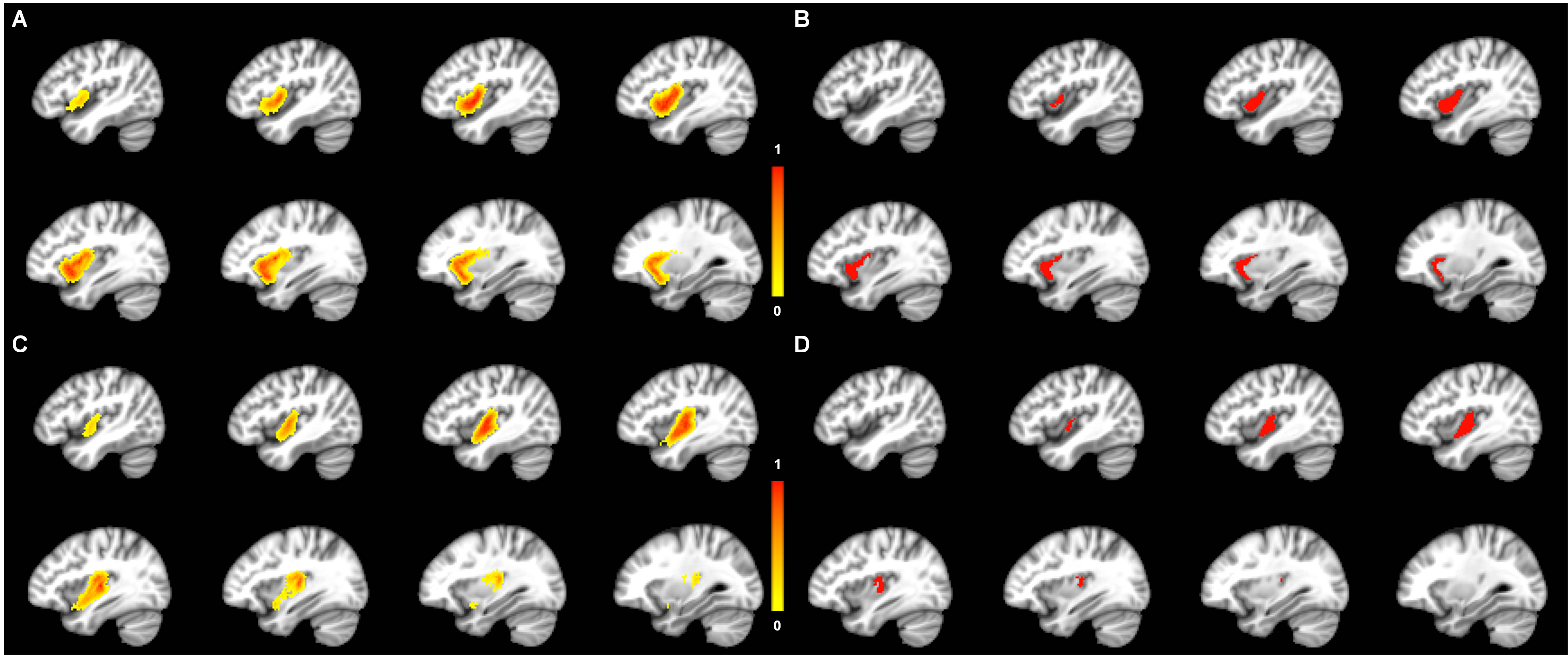

Supplement: S2 Fig — (A) Probability map for the anterior insular cortex from 33 healthy subjects. (B) Anterior insular to ensure 50% probability in (A). (C) Probability map for the posterior insular cortex from 33 healthy subjects. (D) Posterior insular to ensure 50% probability in (C). (TIF) [file pone.0180479.s002.tif]

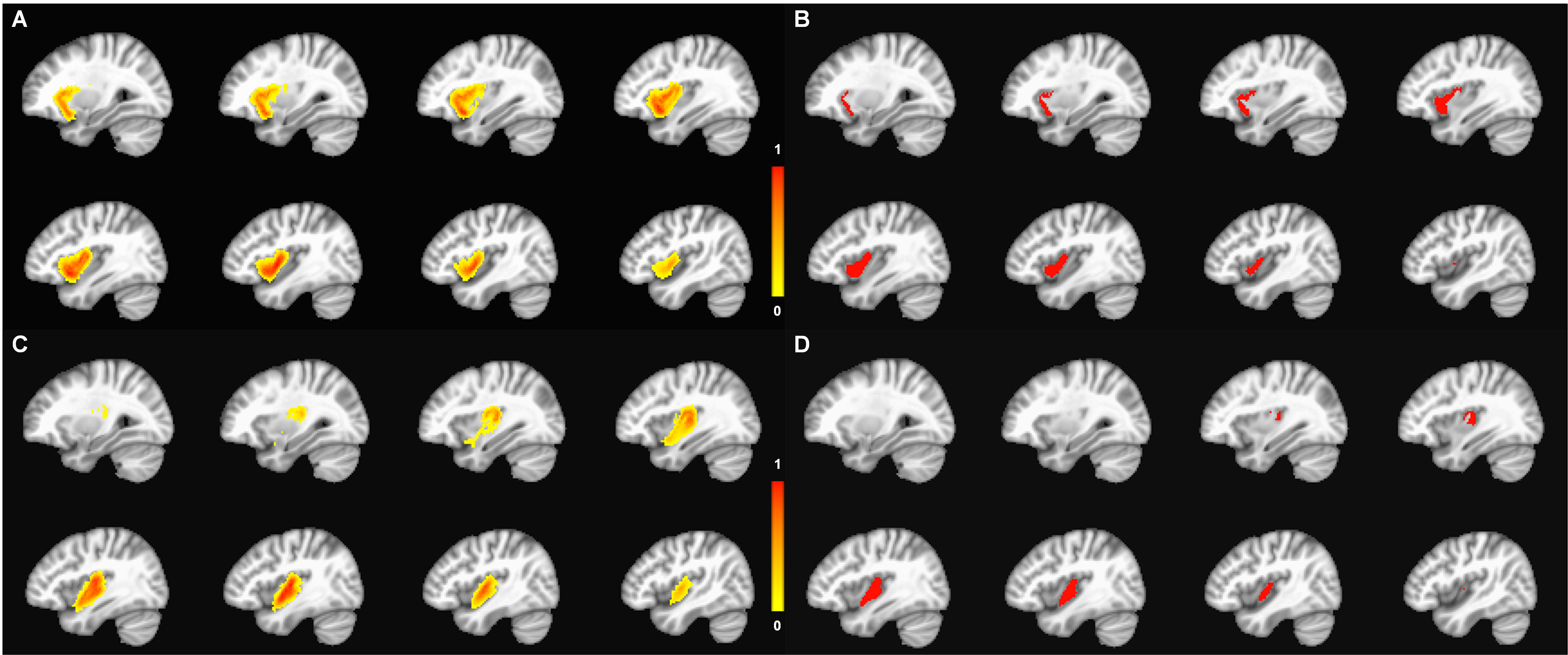

Supplement: S3 Fig — (A) Probability map for the anterior insular cortex from 33 healthy subjects. (B) Anterior insular to ensure 50% probability in (A). (C) Probability map for the posterior insular cortex from 33 healthy subjects. (D) Posterior insular to ensure 50% probability in (C). (TIF) [file pone.0180479.s003.tif]
