# Supplementary material for: Impaired insula functional connectivity associated with persistent pain perception in patients with complex regional pain syndrome
Source: PLoS One. 2017 Jul 10;12(7):e0180479. doi: 10.1371/journal.pone.0180479 (PMC5503260; doi:10.1371/journal.pone.0180479)
Supplement: S1 Table — (DOCX) [file pone.0180479.s004.docx]

**S1 Table. Participant characteristics.**

|  | **Age,**  **year** | **Sex** | **Handed-ness** | **CRPS type** | **Duration,**  **year** | **Pain location** | **Psychiatric comorbidity** | **MPQ**  **sensory** | **MPQ**  **affect** | **MPQ**  **VAS** | **GAF** | **BDI** | **BAI** |
| --- | --- | --- | --- | --- | --- | --- | --- | --- | --- | --- | --- | --- | --- |
| CRPS01 | 36 | M | R | I | 1.0 | Multiple | Other mood disorder | - | - | 7 | 21 | 25 | 36 |
| CRPS02 | 51 | F | R | I | 3.6 | Multiple | Other mood disorder | - | - | 7 | 36 | 52 | 50 |
| CRPS03 | 22 | F | R | I | 2.1 | Multiple | Anxiety disorder | 12 | 8 | 7 | 65 | 16 | 29 |
| CRPS04 | 22 | M | R | I | 1.4 | R lower limb | MDD | 33 | 11 | 8 | 38 | 39 | 38 |
| CRPS05 | 25 | M | R | I | 4.1 | L lower limb | MDD | 33 | 3 | 7.5 | 71 | 12 | 13 |
| CRPS06 | 28 | M | R | II | 0.6 | Multiple^a^ | None | 30 | 12 | 3 | 40 | 47 | 44 |
| CRPS07 | 44 | M | L | II | 1.1 | R lower limb | Other mood disorder | 6 | 3 | 1 | 48 | 36 | 20 |
| CRPS08 | 33 | M | R | I | 2.0 | Multiple | MDD | 15 | 1 | 4 | 43 | 20 | 21 |
| CRPS09 | 30 | F | R | I | 1.4 | Multiple | MDD | 33 | 11 | 7 | 38 | 38 | 47 |
| CRPS10 | 40 | F | R | I | 0.6 | Multiple | None | 7 | 4 | 3 | 75 | 5 | 14 |
| CRPS11 | 20 | M | L | I | 0.6 | L upper limb | None | 22 | 7 | 5 | 65 | 18 | 33 |
| CRPS12 | 33 | F | R | I | 2.0 | Multiple | MDD | 21 | 6 | 6 | 38 | 29 | 18 |
| CRPS13 | 37 | F | R | I | 4.8 | Multiple | Other mood disorder | - | 9 | 8 | 58 | 52 | 53 |
| CRPS14 | 31 | F | R | I | 5.6 | R lower limb | MDD | 21 | 12 | 5 | 34 | 34 | 56 |
| CRPS15 | 59 | F | R | I | 11.4 | Multiple | MDD | 6 | 2 | 4 | 46 | 38 | 16 |
| CRPS16 | 23 | M | R | I | 1.6 | L upper limb | MDD | - | - | 1 | 60 | 35 | 23 |
| CRPS17 | 52 | F | R | I | 0.7 | Multiple | MDD | 14 | 9 | 6 | 40 | 32 | 54 |
| CRPS18 | 20 | M | R | I | 2.9 | R lower limb | None | 23 | 6 | 4 | 78 | 10 | 8 |
| CRPS19 | 53 | F | R | I | 0.3 | Multiple | None | - | - | 0 | 70 | - | - |
| CRPS20 | 49 | F | R | I | 0.2 | Multiple | MDD | 14 | - | 3 | 48 | 28 | 15 |
| CRPS21 | 41 | F | R | I | 0.5 | L upper limb | MDD | 28 | 9 | 4 | 48 | 21 | 22 |
| CRPS22 | 49 | F | R | I | 3.3 | L upper limb | Other mood disorder | 5 | 1 | 3 | 48 | 3 | 3 |
| CRPS23 | 38 | M | R | I | 11.3 | L upper limb | Other mood disorder | 27 | 8 | 7 | 54 | 25 | 16 |
| CRPS24 | 31 | M | R | I | 1.3 | L lower limb | None | 28 | 9 | 7 | 61 | 29 | 40 |
| CRPS25 | 36 | M | R | I | 5.0 | L upper limb | Other mood disorder | - | - | 7 | 55 | - | - |
| HC01 | 26 | M | R | - | - | - | - | - | - | - | - | 8 | 3 |
| HC02 | 29 | F | R | - | - | - | - | - | - | - | - | 0 | 1 |
| HC03 | 27 | M | R | - | - | - | - | - | - | - | - | 0 | 0 |
| HC04 | 25 | F | R | - | - | - | - | - | - | - | - | 0 | 0 |
| HC05 | 25 | M | R | - | - | - | - | - | - | - | - | 7 | 5 |
| HC06 | 25 | M | R | - | - | - | - | - | - | - | - | 0 | 0 |
| HC07 | 25 | M | L | - | - | - | - | - | - | - | - | 3 | 2 |
| HC08 | 28 | M | R | - | - | - | - | - | - | - | - | 4 | 2 |
| HC09 | 34 | F | R | - | - | - | - | - | - | - | - | 2 | 1 |
| HC10 | 37 | F | R | - | - | - | - | - | - | - | - | 5 | 1 |
| HC11 | 24 | F | R | - | - | - | - | - | - | - | - | 3 | 3 |
| HC12 | 35 | M | R | - | - | - | - | - | - | - | - | 0 | 1 |
| HC13 | 35 | M | R | - | - | - | - | - | - | - | - | 0 | 0 |
| HC14 | 40 | F | R | - | - | - | - | - | - | - | - | 1 | 7 |
| HC15 | 26 | M | L | - | - | - | - | - | - | - | - | 2 | 14 |
| HC16 | 24 | M | L | - | - | - | - | - | - | - | - | 3 | 0 |
| HC17 | 31 | F | R | - | - | - | - | - | - | - | - | 4 | 1 |
| HC18 | 32 | M | R | - | - | - | - | - | - | - | - | 4 | 3 |
| HC19 | 34 | F | R | - | - | - | - | - | - | - | - | - | - |
| HC20 | 40 | M | R | - | - | - | - | - | - | - | - | 11 | 1 |
| HC21 | 31 | M | R | - | - | - | - | - | - | - | - | 2 | 1 |
| HC22 | 35 | F | R | - | - | - | - | - | - | - | - | 4 | 3 |
| HC23 | 31 | M | R | - | - | - | - | - | - | - | - | 5 | 2 |
| HC24 | 46 | F | R | - | - | - | - | - | - | - | - | 2 | 1 |
| HC25 | 48 | F | R | - | - | - | - | - | - | - | - | - | - |

Abbreviations: CRPS, complex regional pain syndrome; HC, healthy controls; M, male; F, female; R, right; L, left; MDD, Major depressive disorder; MPQ, McGill Pain Questionnaire; VAS, visual analogue scale; GAF, Global Assessment of Functioning; BDI, Beck Depression Inventory; BAI, Beck Anxiety Inventory; -, not available.

^a^ L upper and lower limbs.
